# Electric Field Assisted Self-Assembly of Viruses into Colored Thin Films

**DOI:** 10.3390/nano9091310

**Published:** 2019-09-13

**Authors:** James J. Tronolone, Michael Orrill, Wonbin Song, Hyun Soo Kim, Byung Yang Lee, Saniya LeBlanc

**Affiliations:** 1 Department of Biomedical Engineering, The George Washington University, Washington, DC 20052, USA; jimtronolone@gmail.com; 2 Department of Mechanical and Aerospace Engineering, The George Washington University, Washington, DC 20052, USA; michael_orrill@gwu.edu; 3 Department of Mechanical Engineering, Korea University, Seoul 02841, Korea; wonbinful@naver.com (W.S.); kenchan888@korea.ac.kr (H.S.K.); blee@korea.ac.kr (B.Y.L.)

**Keywords:** M13 bacteriophage, nanobiomaterial, self-assembly, colorimetric film, electric field, electrowetting

## Abstract

Filamentous viruses called M13 bacteriophages are promising materials for devices with thin film coatings because phages are functionalizable, and they can self-assemble into smectic helicoidal nanofilament structures. However, the existing “pulling” approach to align the nanofilaments is slow and limits potential commercialization of this technology. This study uses an applied electric field to rapidly align the nanostructures in a fixed droplet. The electric field reduces pinning of the three-phase contact line, allowing it to recede at a constant rate. Atomic force microscopy reveals that the resulting aligned structures resemble those produced via the pulling method. The field-assisted alignment results in concentric color bands quantified with image analysis of red, green, and blue line profiles. The alignment technique shown here could reduce self-assembly time from hours to minutes and lend itself to scalable manufacturing techniques such as inkjet printing.

## 1. Introduction

Self-assembly is a strategy in nanofabrication that enables long-range ordering of nanomaterials into highly-organized structures with unique optical, electrical, or magnetic properties [1,2,3,4]. It overcomes limitations in traditional nanofabrication methods such as the need for expensive equipment, high-cost materials, and extensive manufacturing protocols [5,6]. Utilizing this fabrication method, materials including carbon nanotubes, nanoscale metallic particles, and biological nanofilaments can be assembled into thin films for flexible electronics, novel integrated circuits, and sensors [7,8,9,10,11,12]. Using inexpensive nanomaterials, devices fabricated via self-assembly can be manufactured scalably at low cost [6,13]. With the growing need for novel methods that overcome nanofabrication limitations, devices featuring thin films of self-assembled nanomaterials emerge as a commercially viable technology.

**Figure 1:**
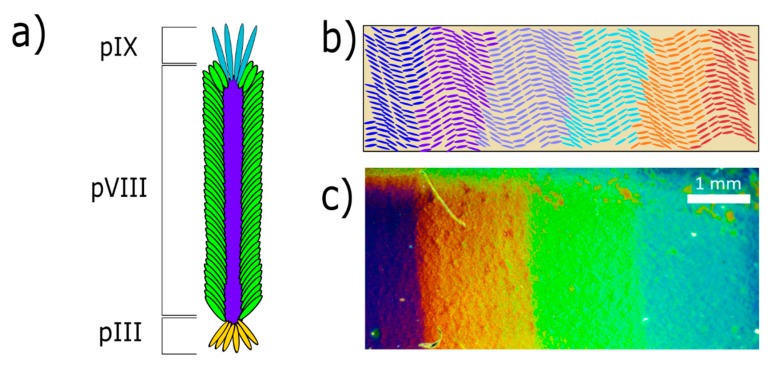
(**a**) A schematic of an individual M13 bacteriophage featuring the filamentous shape with a diameter of approximately 6 nm and a length of 900 nm. An individual phage is coated with approximately 2700 copies of the minor pVIII coat protein, giving the phage a slightly negative surface charge, while fewer pIX and pIII proteins are situated at the head and tail, respectively [22,23]. (**b**) A schematic of a phage litmus with bundles of phage assembled into smectic helicoidal nanofilament (SHN) structures on a substrate. Color change is achieved by the shrinking and swelling of the SHN bundles in response to different compounds binding to the coat proteins. (**c**) An image of a phage litmus fabricated as a pulling method reference sample in this study.

An inexpensive and versatile material used for its ability to self-assemble is M13 bacteriophage [14,15,16,17,18]. Individual bacteriophages, shown in Figure 1a, are bundled and arranged into smectic helicoidal nanofilaments (SHNs) during self-assembly. Additionally, bacteriophages can be genetically engineered, so specific protein motifs can be added to its surface to create binding sites for other molecules. Due to these unique properties, thin films of self-assembled phage SHNs are utilized in a range of devices including phage litmus sensors, rechargeable batteries, and piezoelectric generators [17,19,20]. A schematic illustrating the phage litmus concept is shown in Figure 1b, and Figure 1c shows a fabricated device featuring the characteristic colors of SHN bundles that can be seen by the naked eye [21]. Self-assembled thin films of bacteriophages offer potential for a wide range of commercial applications.

Realizing the potential of self-assembled nanomaterials requires innovations in self-assembly methods. The widely used method of phage assembly is a capillary force-driven coating technique, colloquially termed the “pulling method”. The method involves slowly pulling a small substrate at a 90° angle and at a rate of 20–80 μm/min out of a dispersion of the nanomaterial in deionized (DI) water [15,16,21]. The slow fabrication rate is a manufacturing bottleneck for scalable thin-film manufacturing and commercialization. For instance, coating a 50 mm long substrate at a pulling speed of 80 μm/min would take over 10 h, so a faster method for organizing bacteriophage into SHN structures is needed.

In this work, we increase the rate at which M13 bacteriophage filaments align. The approach induces phage alignment by applying an alternating electric field on sessile evaporating droplets of an M13 bacteriophage dispersion: The electrowetting force applied to the contact line aligns the nanofilaments. The treatment causes a redistribution of charges along the three-phase contact line. The three-phase contact line is the interface of the liquid bacteriophage dispersion, the solid substrate, and the air [24]. This redistribution of charges along this line modifies the interfacial- and line-tension between phases, resulting in an additional force pulling outward on the three-phase contact line, apart from the interfacial tension between liquid–vapor, liquid–solid, and solid–vapor phases [25]. When the amplitude of the applied field is such that the additional force exceeds the magnitude of pinning forces, the three-phase contact line becomes mobile and recedes at a near-constant rate as the droplet evaporates. The recession mimics the pulling method and results in radially aligned SHN structures and concentric color banding. This method is faster than the pulling method and could be implemented in large-scale manufacturing techniques such as inkjet printing.

## 2. Materials and Methods

### 2.1. Nanomaterial Synthesis

The M13 bacteriophage’s major coat proteins (pVIII) were genetically engineered as described previously [26]. Briefly, PCR amplification was performed using Phusion DNA Polymerase, insertion and linearization primers, and an M13KE vector with an engineered PstI site as the template. We prepared two different strains of bacteriophages by displaying two different motifs on the pVIII coat proteins: 4E (*Glu(E)-Glu(E)-Glu(E)-Glu(E)*) and WHWQ (*Trp(W)-His(H)-Trp(W)-Gln(Q)*). The 4E bacteriophage has binding motifs for volatile organic compounds such as hexane, isopropanol, diethyl ether, and methane, and WHWQ strains show selectivity towards aromatic molecules such as toluene [16]. The prepared bacteriophage genomes were purified on an agarose gel, eluted by spin column purification, digested with PstI, and recircularized by overnight ligation at 16 °C with T4 DNA ligase. The ligated DNA vector was transformed into XL1-Blue electroporation competent *Escherichia coli* bacteria (Agilent Technologies, Santa Clara, CA, USA). The constructed phages were amplified using bacterial cultures and purified through standard polyethylene glycol precipitation using polyethylene glycol with an average molecular weight of 4000 (Polyscience Inc., Warrington, PA, USA). The phage dispersion was further purified by filtration through 0.45 µm pore size membranes. To verify phage stability, the amplified plasmid sequence was verified with a sequencing service (Cosmo Genentech, Seoul, Korea). Concentrations of phage dispersions in DI-water were adjusted to 2, 4, and 6 mg/mL. These dispersions were used later for studying the effects of phage concentration on the quality of color change. A 5.5 mg/mL concentration dispersion was also used in the experiments investigating electric field impacts.

### 2.2. Substrate Preparation

Substrates were prepared by dicing gold-coated silicon wafers (150 nm thick gold film thermally evaporated on a 500 µm thick p-doped Si wafer) and silicon dioxide (SiO_2_) wafers (University Wafer, 300 nm wet thermal oxide) into 1 cm × 1 cm squares. The wafers were cleaned sequentially in ultrasonic baths of acetone, methanol, isopropyl alcohol, and DI water for 8 min each and then dried with compressed air. Gold-coated silicon substrates were used because the 4E and WHWQ strains of M13 bacteriophages used in this experiment featured binding motifs for gold [16,21,27]. Silicon dioxide substrates were used to eliminate Joule heating due to current flow under the droplet on the gold substrates between electrodes.

To prepare a reference sample, a gold-coated silicon wafer substrate was dipped into a bacteriophage dispersion (5 mg/mL in DI water) and pulled out slowly. Color bands on the substrate were controlled using different gradual pulling speeds from 20 to 100 μm/min by a programmable syringe pump (LEGATO270, KD Scientific Co., Holliston, MA, USA).

A shadow mask with multiple 1 mm × 5 mm rectangular holes separated by a space of 5 mm was used to create pairs of parallel-plate silver electrodes via thermal evaporation (Denton DV-202A, Moorestown, NJ, USA). A copper boat holding silver shot (Kamis Inc., Mahopac Falls, NY, USA, 99.99% + pure) was placed in the evaporator chamber, which was pumped down to 20 mTorr. The current was increased in steps of 10 A up to 70 A with a soak time of 3 min. The current was then held at 70 A until the crystal monitor (Standard 6 MHz gold, Kurt J. Lesker Company, Jefferson Hills, PA, USA) indicated a deposition height of 2 µm.

### 2.3. Electric Field Application

**Figure 2:**
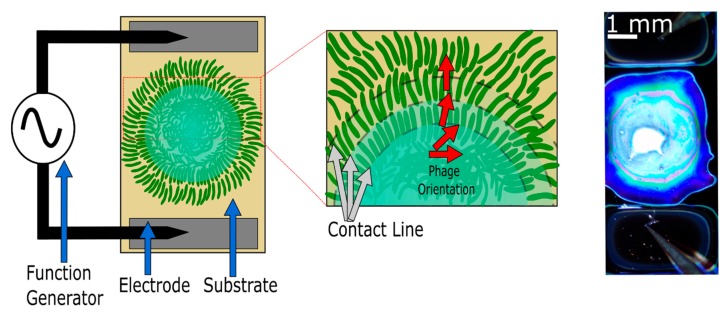
Schematic of the experimental setup. The applied field causes charges to redistribute along the three-phase contact line, modifying the interfacial- and line-tension between phases, causing the contact line to become unpinned from the substrate and recede at a near-constant rate as the droplet evaporates. As the contact line recedes, the phage bundles become radially aligned. The red arrows demonstrate the orientation of aligning bacteriophages. The dashed line represents the contact line.

Electrowetting experiments were carried out in a 2-point probe station. Figure 2 shows a diagram of the experimental setup with a schematic of the bacteriophage alignment following droplet evaporation. Probes were placed onto the electrodes to apply the alternating electric field while images and videos of the evaporating bacteriophage droplets were recorded. The applied voltage and frequency were chosen based on previous work that immobilized and aligned bacteriophages [28,29,30]. In this work, we used voltages of 5, 10, 15, and 20 V_pp_ at 15 kHz. Droplets of 10 μL evaporated between the electrodes in the presence of an applied electric field.

We studied both the effect of varying bacteriophage concentration and applied electric field on SHN structure alignment. Bacteriophages dispersed in DI water at concentrations of 2, 4, and 6 mg/mL were drop-cast onto substrates with 10 V_pp_ applied across gold-coated substrates. Voltages of 5, 10, 15, and 20 V_pp_ were applied across gold-coated and SiO_2_-coated substrates with droplets containing a dispersion of bacteriophages at a concentration of 5.5 mg/mL.

### 2.4. RGB Color Characterization

Video recordings of evaporating droplets and images of the resulting deposits were obtained by using a microscope (Omano Microscope OM2300S-V14, The Microscope Store, Roanoke, VA, USA) equipped with a recording camera (Summit Camera SK2-10X, The Microscope Store, Roanoke, VA, USA). The colored-film quality of all samples was observed using a high magnification microscope (DM2700M, Leica, Wetzlar, Germany) equipped with a camera (MC-190, Leica, Wetzlar, Germany). To quantify the difference in appearance of color banding between the resulting deposition of the electric field-treated and untreated samples, images of the concentric color bands were analyzed. One-dimensional radial color (RGB) profiles were measured from the center of each deposit to the edge with the ImageJ plugin RGBProfiler. The raw RGB pixel intensity data was smoothed in MATLAB with a running average filter (smooth, span of 63). The derivative of pixel intensity with respect to the normalized radius was calculated in Python (numpy.gradient, version 3.7, Python Software Foundation, DE, USA).

### 2.5. AFM Analysis

Atomic force microscopy (MFP-3D AFM, Asylum Research, Goleta, CA, USA) was performed on electric field-treated and untreated samples to observe the alignment of bacteriophages. Areas of 50 µm^2^ were scanned at 0.10 Hz with standard silicon cantilevers (AC160TS R3, Asylum Research, Goleta, CA, USA) using the tapping mode with a tip spring constant of 26 N/m and resonance frequency of 305 kHz. Images were exported and edited using Igor Pro 6.3.7.2 (Asylum Research, Goleta, CA, USA).

## 3. Results and Discussion

**Figure 3:**
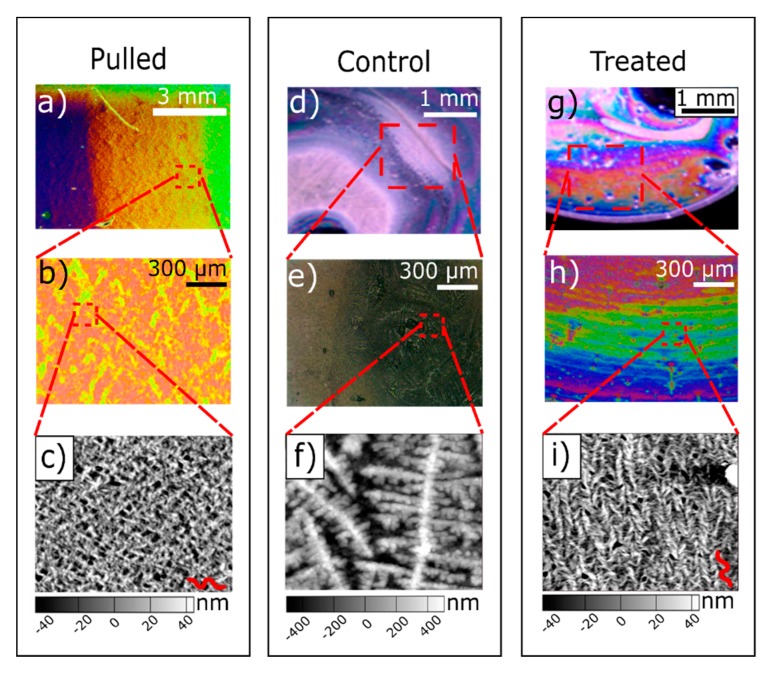
Optical and atomic force microscope images of reference, control, and experimental samples. (**a**) Reference sample fabricated by the pulling method. (**b**) Magnified section from the pulled sample, showing a film of SHN bacteriophage reflecting a single color. (**c**) Atomic form microscope (AFM) image of sample exhibiting SHN structures fabricated by the puling method. An SHN is traced in red to help identify the structure. (**d**) Control sample of a bacteriophage dispersion drop-cast with no applied electric field. (**e**) Magnified section from control sample, exhibiting no color change. (**f**) AFM image of untreated control sample exhibiting agglomerated bacteriophages. (**g**) Electric field-treated sample using a 6 mg/mL M13 bacteriophage dispersion treated with 10 V_pp_ at 15 kHz during evaporation. (**h**) Magnified section of the electric field-treated sample, exhibiting a color change due to the SHN structure but with shorter and less uniform color bands compared to 3b. (**i**) AFM image of the electric field-treated sample showing aligned bundles of bacteriophages in SHN structure. A tracing of one of the SHNs is shown in red to help identify the bacteriophage bundle structure.

Distinct concentric color banding occurred in deposits of M13 bacteriophage droplets that evaporated in the presence of an alternating electric field (Figure 3). No color banding occurred in the deposits of untreated control samples. During evaporation, an applied AC electric field induces an electrowetting force that reduces the contact angle and pulls outward on the three-phase contact line [31]. The magnitude of the electrowetting force oscillates with the applied field, and periodically exceeds pinning forces between defects on the substrate surface and the three-phase contact line, allowing the contact line to move freely [25]. This periodic slip-stick motion resulted in near-constant recession of the contact line during droplet evaporation that promoted the self-assembly of M13 bacteriophage SHN structures similar to the films produced by the pulling method [16,21]. Both electric field-treated and untreated control samples exhibited a thick film of M13 bacteriophage deposited at the droplet center that increased in size with increasing phage concentration. No trend was observed for varying applied voltage, phage concentration, or substrate type.

Reference and control samples were compared with electric field-treated samples. The reference samples were M13 bacteriophage films on gold substrates obtained with the pulling method at various pull rates (20, 40, 60, and 80 µm/min), shown in Figure 3a–c [16,21]. The film quality and uniformity of this reference sample is representative of requirements for commercially viable thin films of M13 bacteriophages. The control samples are drop-cast M13 bacteriophage droplets that evaporated without an applied electric field on both substrate types, SiO_2,_ and gold.

Figure 3 shows droplets that evaporated in the presence of an electric field exhibited wide bands of color towards the outside of the droplet (Figure 3g) while control samples showed no color throughout the sample (Figure 3d). Under higher magnification, control samples exhibited dark gray to black colors, and electric field-treated samples exhibited distinct colors in concentric bands along the outer regions of deposits. Periodic color banding decreased as the phage concentration remaining in the DI water became too high for controlled particle alignment on the substrate; the high phage concentration disrupts the balance of attractive and repulsive forces between bacteriophages, thus inhibiting formation of an ordered thin film of SHN bundles.

**Figure 4:**
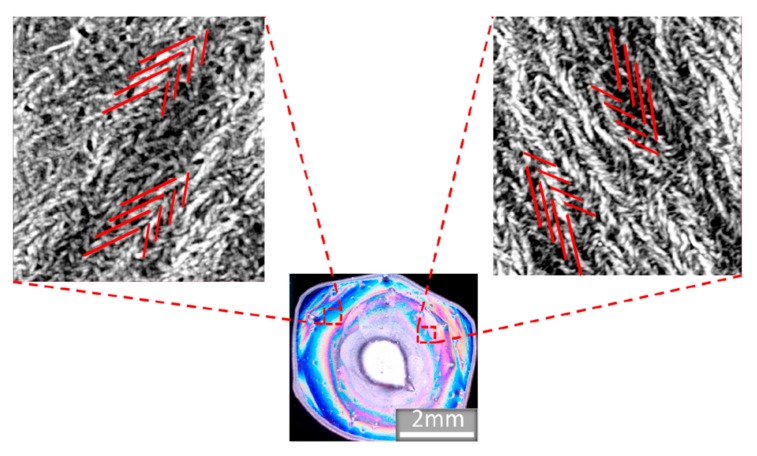
AFM images taken at different points along the radial color bands. Red lines representing SHN structure are drawn over AFM images to aid in visualizing the direction of the SHN bundles. Each line represents many bacteriophages bundled together to create building blocks of the self-assembled SHN film.

Atomic force microscope (AFM) images confirmed that color banding was due to the presence of SHN structures. Aligned M13 bacteriophage SHN structures produced by the pulling method (Figure 3c) were compared with the film structure of drop-cast M13 bacteriophage droplets that evaporated with (Figure 3i) and without (Figure 3f) an applied electric field. The film thickness resulting from the pulling method and the electric field-treated samples were similar; both showed aligned SHN structures. The film from the drop-cast control sample (Figure 3f) showed large agglomerations of M13 bacteriophage bundles in random orientation with a film thickness that is 10 times that of the electric field-treated and pulled samples. AFM images displayed in Figure 4 further show the radial color bands in experimental samples were due to SHN alignment in the radial direction.

**Figure 5:**
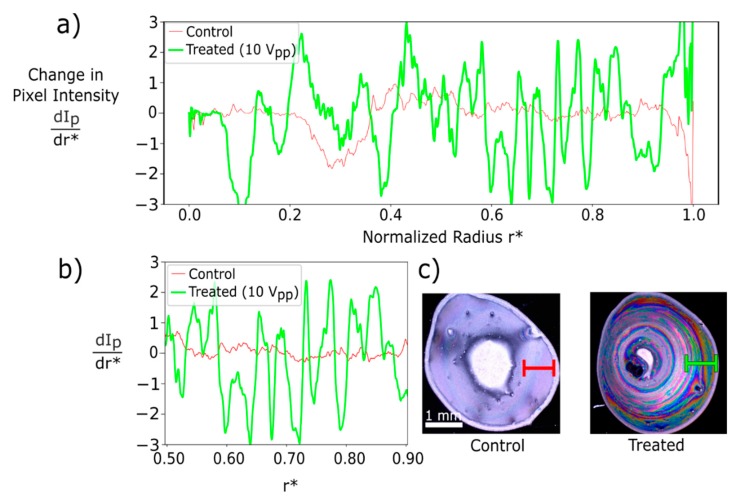
(**a**) Derivative of pixel intensity ($\frac{\mathrm{dIp}\;}{\mathrm{dr}*}$) over the normalized radius (r*) for a control and electric field-treated (10 V_pp_) sample. The control signal shows only slight variation, with values close to or at zero, indicating little to no color banding. The experimental signal shows high variation throughout the deposit region indicating a high degree of color banding. (**b**) ($\frac{\mathrm{dIp}\;}{\mathrm{dr}*}$) from 0.5 < r* < 0.9 highlighting the difference between the control and experimental samples where color banding is seen. (**c**) Images of control and experimental samples with overlays showing the line profile over the region 0.5 < r* < 0.9.

Figure 5 presents an example of the derivative of RGB intensity line profiles as a function of the normalized radius, r*, of M13 bacteriophage deposits in control and experimental samples. Without an applied voltage, deposits exhibited little color change: The derivative was mostly flat beyond r* = 0.4, which approximately bounds the thick-film in the center of the deposit. Comparatively, derivatives taken from electric field-treated samples varied significantly in the color-banded region, 0.5 < r* < 0.9 (Figure 5a). Each treated sample showed high variation throughout the color intensity line profile, indicating repeatability of the color-banding result.

Analyzing the derivative of color intensity line profiles was also used to determine the effects of varying the applied electric field strength and the bacteriophage concentration. In the control sample (Supplementary Figure S1a), a droplet with a bacteriophage concentration of 5.5 mg/mL was drop-cast without an applied electric field, resulting in no color change. The derivative of the line profile was zero, indicating no color variation along r*. Distinct color bands were observed in droplets of concentration 2, 4, and 6 mg/mL when exposed to an electric field during evaporation (Supplementary Figure S1b–d). Higher phage concentrations resulted in larger areas of unstructured thick film in the center of the deposit, but concentration had no observable effect on the color bands produced. No trend was revealed by the color intensity line profile analysis.

Using a constant bacteriophage concentration (4 mg/mL), the impacts of increasing the applied voltage and changing substrates were investigated (Supplementary Figure S2). Electric field-treated samples on SiO_2_ substrates showed wide blue and green bands outside of the center of the deposit where a thick film formed. Electric field-treated samples on gold substrates likewise resulted in wide bands of blue at the outer edge of the droplet and a thick film in the center. When performing the RGB analysis, no repeatable trend in color banding was observed with varying applied voltage or different substrates.

Electric field-treated samples on SiO_2_ substrates were further compared to those on gold substrates to isolate any effect of Joule heating caused by current flow between electrodes (Supplementary Figure S2). When using the same experimental setup shown in Figure 2, there was no observable difference in the color-banding or microscopic structures between treated samples on the two substrates.

**Figure 6:**
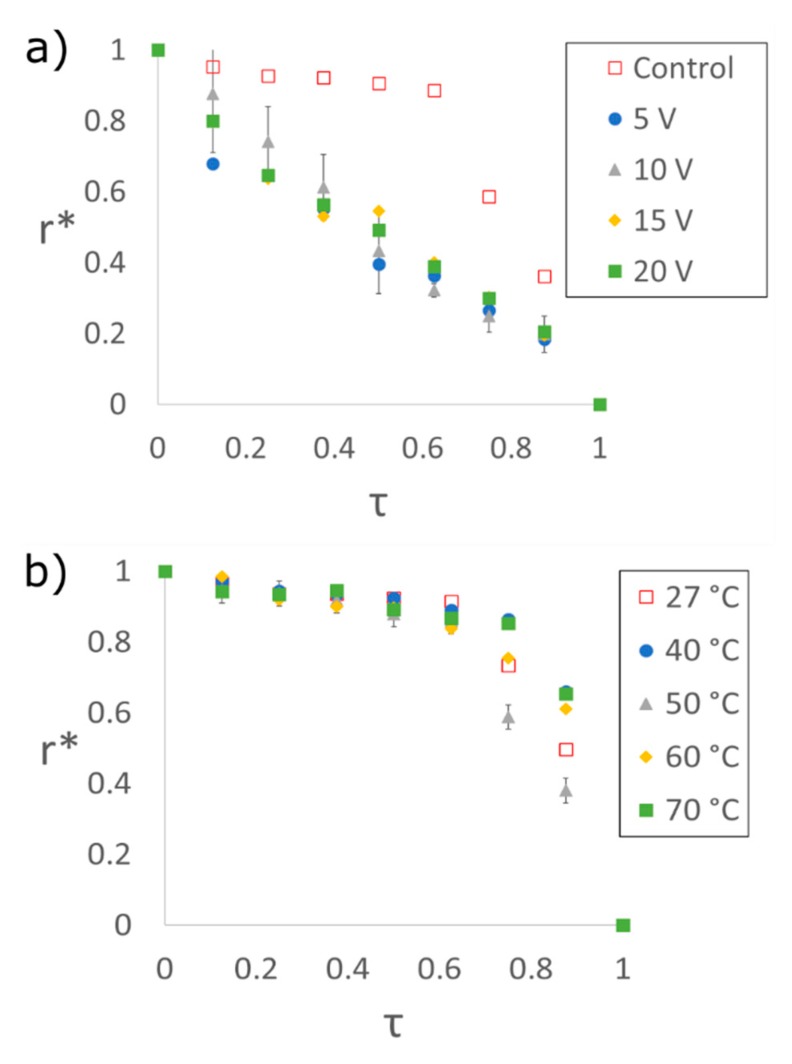
Contact line recession shown as the measured normalized radius (r*) to the edge of an evaporating droplet over the normalized evaporation time (τ). (**a**) Control and treated samples using 5.5 mg/mL M13 bacteriophage dispersion under increasing voltage application. For the control sample, r* is nearly constant up to τ = 0.6 indicating a pinned contact line. With an applied voltage, the contact line moves at a near-constant rate, allowing for the self-assembly of SHNs. (**b**) Contact line recession of droplets on heated substrates with no applied field. Each of the measurements illustrates that Joule-heating alone does not unpin the contact line and allows it to recede as is seen with an applied voltage. Error bars in each plot are shown for the condition with the highest error.

To confirm the contact line becomes unpinned by the application of an electric field, its displacement was measured throughout evaporation and compared to that of droplets with no electric field treatment [32]. Figure 6 shows movement of the contact line via its position normalized by the size of the entire droplet, r*, as a function of time normalized by each sample’s total evaporation time, τ. For untreated droplets, the contact line remained pinned and immobile for the first half of the total evaporation time, whereas the contact line for electric field-treated droplets receded at a near-constant rate throughout the total evaporation time. Performing linear regressions on each set of data demonstrated that applying an electric field increased the uniformity of the contact line recession by 30%. Further, treated samples demonstrated R-squared values of 0.95 or greater, indicating near-uniform recession of the contact line: The electric field forces the contact line to overcome substrate pinning and leads to better phage alignment. Further, deposits resulting from untreated, heated samples showed no improvement in contact line movement (Figure 6b) nor color–color banding, which suggests that the observed SHN structures result solely from the application of an electric field.

These experiments collectively show that application of an AC electric field to an evaporating droplet of M13 bacteriophage causes the contact line to become unpinned and recede at a near-constant rate, mimicking the motion of the meniscus in the pulling method. M13 bacteriophage molecules adsorb to the substrate and form SHN structures aligned in the radial direction, parallel to the direction of contact line motion, resulting in concentric rings of color bands. This new technique allows for rapid phage self-assembly, exhibiting distinct color banding within five minutes. Experiments with varied phage concentration, electric field strength, and substrate type yielded no discernable improvements in film quality. In order to improve film quality, suggested future improvements include influencing the movement of the contact line to produce wider and more uniform color bands. Additionally, optimization of the concentration of bacteriophages dispersed in DI water could result in less thick film deposition in the center of the droplets, promoting a wider color-banded region.

## 4. Conclusions

This work shows an alternating electric field can align M13 bacteriophage nanostructures in evaporating dispersions, enabling thin films of phage SHNs where film color corresponds to phase alignment. This alignment approach is faster than the standard pulling method: By using an alternating electric field, phage structures that show color change can be achieved in minutes as opposed to the hours required for the pulling method. Scalable manufacturing using this approach is feasible and could be implemented in manufacturing processes such as drop-on-demand printing. The results presented here support the viability of rapid, scalable fabrication of devices using M13 bacteriophages.

## Supplementary Materials

The following are available online at https://www.mdpi.com/2079-4991/9/9/1310/s1, Figure S1: Color Change with Respect to Bacteriophage Concentration, Figure S2: Color Change with Respect to Electric Field Strength.
