# Supplementary material for: Electric Field Assisted Self-Assembly of Viruses into Colored Thin Films
Source: Nanomaterials (Basel). 2019 Sep 13;9(9):1310. doi: 10.3390/nano9091310 (PMC6781059; doi:10.3390/nano9091310)
Supplement: Supplementary file 1 [file nanomaterials-09-01310-s001.pdf]

nanomaterials

Electric Field Assisted Self-Assembly of Viruses into Colored Thin Films
Supplementary Information

Figure S1 presents the results of increasing M13 bacteriophage concentration while applying a 10 V_{pp} electric field. The control sample, prepared in the absence of an applied electric field, does not show color variation. The color variation obtained in experimental samples subjected to an applied voltage is thus due to the applied field. While color change was observed in the experimental samples, RGB analyses showed that there was no discernible trend in the thickness of the color bands as concentration of bacteriophages increased. Additionally, the unstructured deposit in the center of the droplet became larger as the concentration increased.

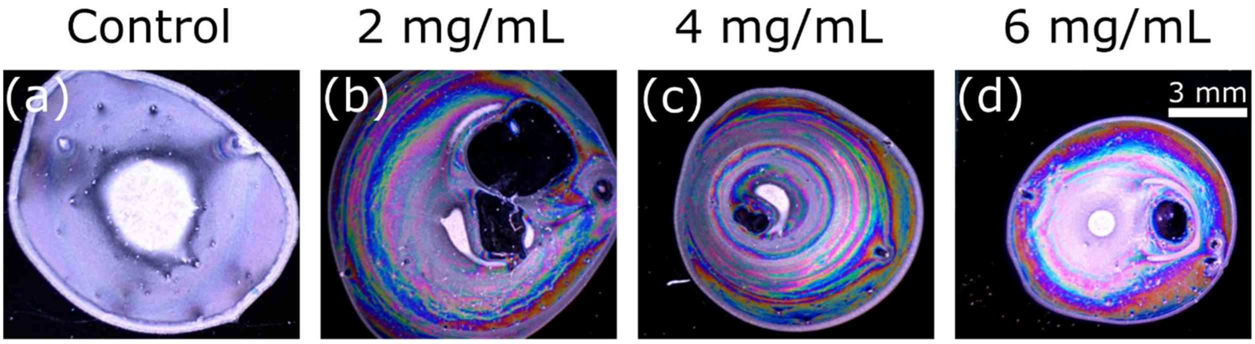

Figure S1. (a) Control sample using 5.5mg/mL bacteriophage solution with no applied electric field demonstrating no color change at any point within the sample. (b) – (d) Samples treated with an applied voltage of 10Vpp at 15kHz, all demonstrating color change after treatment. (b) 2 mg/mL. (c) 4 mg/mL. (d) 6 mg/mL.

When investigating the effect of electric field strength on the ability to achieve color, no trend was seen (Figure S2). RGB analyses also revealed no discernable trend in color band thickness or uniformity as voltage increased. Additionally, color was observed on both gold- and SiO_2-coated substrates, indicating that Joule heating was not solely responsible for the ability to achieve color variations in samples receiving the applied field treatment.

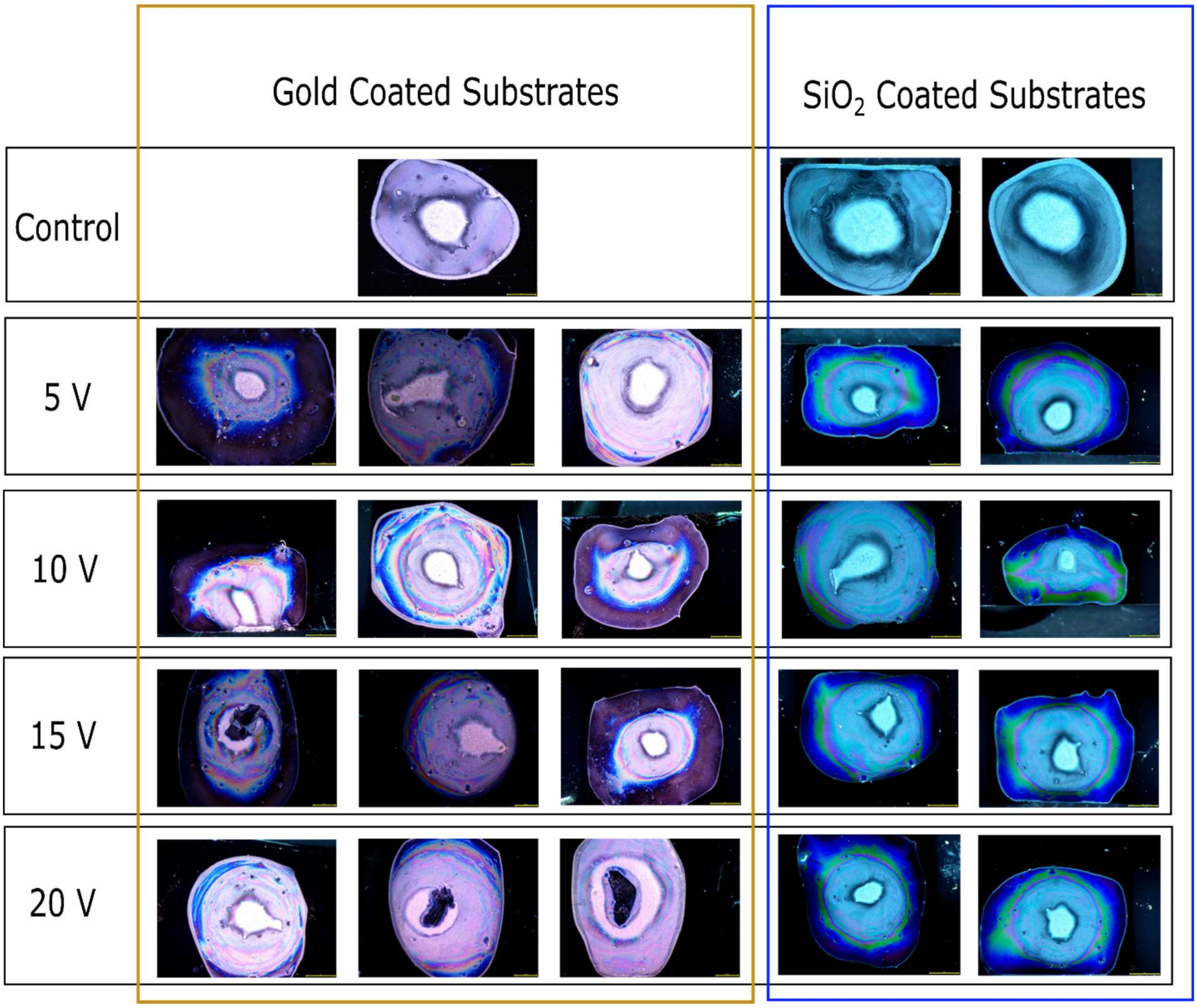

Figure S2. Array of samples arranged by increasing electric field strength. The left three columns are samples using a gold-coated substrate, and the right two columns are samples using a SiO2 coated substrate.
